# Ultrastructure in Transthyretin Amyloidosis: From Pathophysiology to Therapeutic Insights

**DOI:** 10.3390/biomedicines7010011

**Published:** 2019-02-05

**Authors:** Haruki Koike, Masahisa Katsuno

**Affiliations:** Department of Neurology, Nagoya University Graduate School of Medicine, Nagoya 466-8550, Japan; ka2no@med.nagoya-u.ac.jp

**Keywords:** angiopathy, diflunisal, electron microscopy, oligomers, pathogenesis, pathology, protein misfolding disease, Schwann cell, tafamidis, therapy

## Abstract

Transthyretin (TTR) amyloidosis is caused by systemic deposition of wild-type or variant amyloidogenic TTR (ATTRwt and ATTRv, respectively). ATTRwt amyloidosis has traditionally been termed senile systemic amyloidosis, while ATTRv amyloidosis has been called familial amyloid polyneuropathy. Although ATTRwt amyloidosis has classically been regarded as one of the causes of cardiomyopathy occurring in the elderly population, recent developments in diagnostic techniques have significantly expanded the concept of this disease. For example, this disease is now considered an important cause of carpal tunnel syndrome in the elderly population. The phenotypes of ATTRv amyloidosis also vary depending on the mutation and age of onset. Peripheral neuropathy usually predominates in patients from the conventional endemic foci, while cardiomyopathy or oculoleptomeningeal involvement may also become major problems in other patients. Electron microscopic studies indicate that the direct impact of amyloid fibrils on surrounding tissues leads to organ damage, whereas accumulating evidence suggests that nonfibrillar TTR, such as oligomeric TTR, is toxic, inducing neurodegeneration. Microangiopathy has been suggested to act as an initial lesion, increasing the leakage of circulating TTR. Regarding treatments, the efficacy of liver transplantation has been established for ATTRv amyloidosis patients, particularly patients with early-onset amyloidosis. Recent phase III clinical trials have shown the efficacy of TTR stabilizers, such as tafamidis and diflunisal, for both ATTRwt and ATTRv amyloidosis patients. In addition, a short interfering RNA (siRNA), patisiran, and an antisense oligonucleotide (ASO), inotersen, have been shown to be effective for ATTRv amyloidosis patients. Given their ability to significantly reduce the production of both wild-type and variant TTR in the liver, these gene-silencing drugs seem to be the optimal therapeutic option for ATTR amyloidosis. Hence, the long-term efficacy and tolerability of novel therapies, particularly siRNA and ASO, must be determined to establish an appropriate treatment program.

## 1. Introduction

Transthyretin (TTR) amyloidosis is caused by systemic deposition of wild-type or variant amyloidogenic TTR (ATTRwt and ATTRv, respectively). ATTRwt amyloidosis has been traditionally named senile systemic amyloidosis because postmortem studies revealed that its prevalence becomes higher as age at examination increases [1]. On the other hand, ATTRv amyloidosis has been called familial amyloid polyneuropathy [2,3,4,5]. Although this disease was originally reported in geographically restricted areas (i.e., endemic foci) of Portugal, Japan, and Sweden [6,7,8], its global prevalence has been demonstrated [2,9]. The Val30Met mutation, alternatively called p.Val50Met according to the Human Genome Variation Society nomenclature, has been considered the most common mutation because patients from endemic foci and many of the late-onset (more than 50 years of age) patients from nonendemic areas have this mutation [2,10]. However, recent progress in diagnostic techniques has increased the number of newly diagnosed patients with non-Val30Met mutations [11]. Over 130 mutations have been reported so far [12], and certain types of non-Val30Met patients are more frequent than Val30Met patients in some countries [13,14,15].

Regarding the treatment for ATTR amyloidosis, the efficacy of liver transplantation, which is usually indicated for early-onset ATTRv amyloidosis patients, has been established since the 1990s [16,17]. Recent phase III clinical trials have shown the efficacy of TTR stabilizers for both ATTRwt and ATTRv amyloidosis patients [18,19,20]. In addition, gene-silencing drugs that significantly reduce the amount of TTR produced in the liver have also become available for ATTRv amyloidosis [21,22]. Eliminating causative proteins is more reasonable than merely stabilizing the protein because nonfibrillar TTR may also exert harmful effects, as described later. 

In this review, we describe the pathophysiological aspects of ATTR amyloidosis, focusing on the fine structures of amyloid fibrils and their impact on neighboring tissues and therapeutic insights from pathology.

## 2. Mechanisms of Amyloid Deposition

ATTR amyloidosis is a gain-of-toxic-function protein misfolding disease in which variant TTR assembles into amyloid fibrils in extracellular spaces, leading to systemic organ dysfunction. TTR is a 55-kD homotetrameric protein composed of 127-residue β-sheet-rich subunits [23]. Although TTR protein is mainly synthesized in the liver, production also occurs at other sites, such as the choroid plexus in the brain and the retinal pigment epithelium in the eye [24,25]. TTR produced in the liver is responsible for the major manifestations of ATTR amyloidosis, such as neuropathy and cardiomyopathy. By contrast, TTR produced by the choroid plexus and retinal pigment epithelium may also cause oculoleptomeningeal amyloidosis [26].

TTR is stable in its homotetramer form and functions as a transporter of thyroxin (T4) and retinol (vitamin A)-binding protein under physiological conditions [27,28]. It is widely accepted that the dissociation of natively folded TTR tetramers into monomers is a crucial step in the disease process, particularly in the aggregation of amyloid fibrils in ATTR amyloidosis [29,30]. Most *TTR* mutations result in the production of TTR that is less stable than wild-type TTR, leading to aggressive and systemic amyloid deposition of variant TTR [30]. The dissociation and subsequent aggregation of TTR may occur even in subjects without *TTR* mutations in certain conditions, such as aging, leading to an occurrence of ATTRwt amyloidosis [31].

In addition to this TTR tetramer dissociation and the subsequent misfolding pathway, recent studies suggested the presence of an alternative pathway associated with proteolytic cleavage of TTR during the process of amyloid fibril formation, as described later [32,33]. 

## 3. Diversity of Clinical Features

As ATTR amyloidosis is a systemic disease, patients exhibit variable clinical features depending on the site of amyloid deposition [34]. ATTRwt amyloidosis has classically been regarded as one of the causes of cardiomyopathy in the elderly population. Studies of autopsy specimens revealed that a significant proportion of the elderly population have wild-type TTR deposition, particularly in the heart (12 to 25% of subjects aged >80 years), despite a lack of relevant symptoms [35,36,37]. However, the recent development of diagnostic techniques for amyloidosis has significantly expanded the concept of this disease [38]. For example, this disease is now considered an important cause of carpal tunnel syndrome in the elderly population [38,39]. Some studies have also suggested an association between wild-type TTR deposition in ligaments and spinal canal stenosis [38,40,41].

The phenotypes of ATTRv amyloidosis are also variable, depending on the mutation and age at onset [2,12]. As the classical name “familial amyloid polyneuropathy” indicates, peripheral neuropathy usually predominates in patients with conventional endemic foci [42,43]. Cardiomyopathy or oculoleptomeningeal involvement may also become major problems in others, particularly in patients with non-Val30Met mutations [12,44]. For example, Val112Ile and Thr60Ala mutations are usually associated with cardiac amyloidosis, while Tyr114Cys mutation causes oculoleptomeningeal amyloidosis [12]. Regarding the most common mutation, Val30Met (i.e., ATTR Val30Met amyloidosis), patients from the conventional endemic foci of Portugal and Japan exhibit textbook features of amyloid neuropathy, such as the following: early disease onset ranging in age from the late 20s to early 40s; a high penetrance rate; a nearly 1-to-1 male-to-female ratio; marked autonomic dysfunction; loss of superficial sensation, including nociception and thermal sensation (i.e., sensory dissociation); atrioventricular conduction block requiring pacemaker implantation; and the presence of anticipation of age at onset (Table 1) [2,45,46,47]. By contrast, patients with Val30Met mutations from nonendemic areas exhibit an older age at disease onset of over 50 years, a low penetrance rate, extreme male preponderance, relatively mild autonomic dysfunction, loss of all sensory modalities rather than sensory dissociation, the frequent presence of cardiomegaly, and the absence of anticipation of age at onset [2,10,48,49,50]. Despite the presence of the same mutation in the *TTR* gene, the reason for the differential clinical features between early- and late-onset cases has not been clarified.

## 4. Pathological Findings Corresponding to Clinical Characteristics

**Figure 1:**
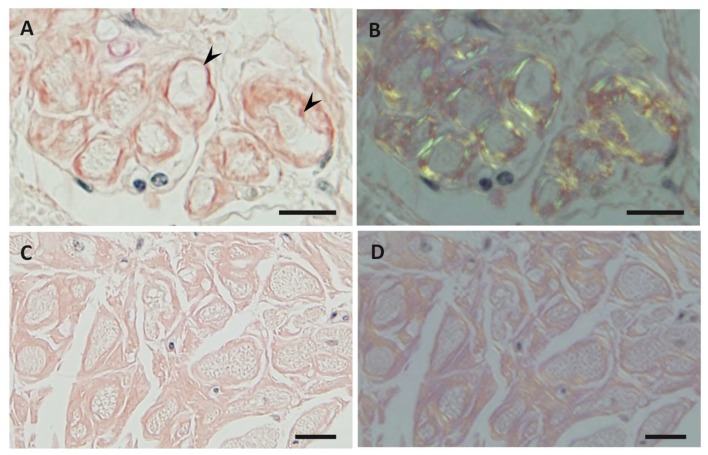
Representative photographs of cardiac amyloid deposits in early-onset ATTR Val30Met amyloidosis patients from endemic foci (**A**,**B**) and late-onset ATTR Val30Met amyloidosis patients from nonendemic areas (**C**,**D**) obtained at autopsy. Alkaline Congo red staining. In early-onset patients from endemic foci, the amyloid deposits tend to be highly congophilic (**A**) and show strong apple-green birefringence (**B**). In addition, amyloid deposits tend to induce atrophy and degeneration of myocardial cells, particularly in the subendocardial layer, producing a histologic picture of amyloid rings (arrowheads). In late-onset patients from nonendemic areas, the amyloid deposits are generally weakly congophilic (**C**) and show faint apple-green birefringence (**D**). Atrophy or degeneration of myocardial cells is not conspicuous in late-onset patients from nonendemic areas compared to early-onset patients from endemic foci. Scale bars = 20 μm.

It has been widely accepted that amyloid deposition causes organ dysfunction in ATTR amyloidosis. Hence, it is presumed that the amount of amyloid deposits and the effect of amyloid on surrounding tissues in individual organs determine the phenotype of this disease. For example, cardiac manifestations of ATTR Val30Met amyloidosis are different between conventional early-onset patients from endemic foci and late-onset patients from nonendemic areas [34]. In early-onset cases, cardiac amyloid deposits tend to be found in the atrium and subendocardial region, resulting in the atrophy and degeneration of myocardial cells in the subendocardial layer, producing a histologic picture of amyloid rings (Figure 1A,B) [34]. This alteration in myocardial cells may be related to cardiac conduction abnormalities that frequently occur in early-onset cases [2]. In contrast, amyloid deposition tends to be prominent throughout the layers of myocardium without atrophy or degeneration of myocardial cells in late-onset cases (Figure 1C,D). Accordingly, cardiac enlargement due to massive amyloid deposition leading to diastolic dysfunction rather than cardiac conduction abnormalities is the characteristic feature of late-onset cases [10,34]. Interestingly, the characteristics of cardiomyopathy in late-onset ATTR Val30Met amyloidosis cases are similar to those in ATTRwt amyloidosis cases [52].

Neuropathic features also correspond to pathological alterations in patients with ATTRv amyloidosis. For example, small-fiber-predominant axonal degeneration characterizes conventional early-onset Val30Met cases in endemic foci, whereas both small and large fibers are affected in late-onset Val30Met cases in nonendemic areas (Table 1) [34,51]. These pathological characteristics are, respectively, in accordance with sensory dissociation in early-onset cases and loss of all sensory modalities in late-onset cases [2]. As described later, the predominant loss of small-diameter nerve fibers in early-onset cases is attributable to direct damage by amyloid fibrils that form around nerve fibers. Regarding the late-onset cases, the amount of amyloid deposits is less than that in the early-onset cases for the severity of nerve fiber loss [34]. Further studies are needed to clarify the mechanisms of nerve fiber degeneration in late-onset cases.

## 5. Characteristics of Amyloid Fibrils Determining the Clinicopathological Features

**Figure 2:**
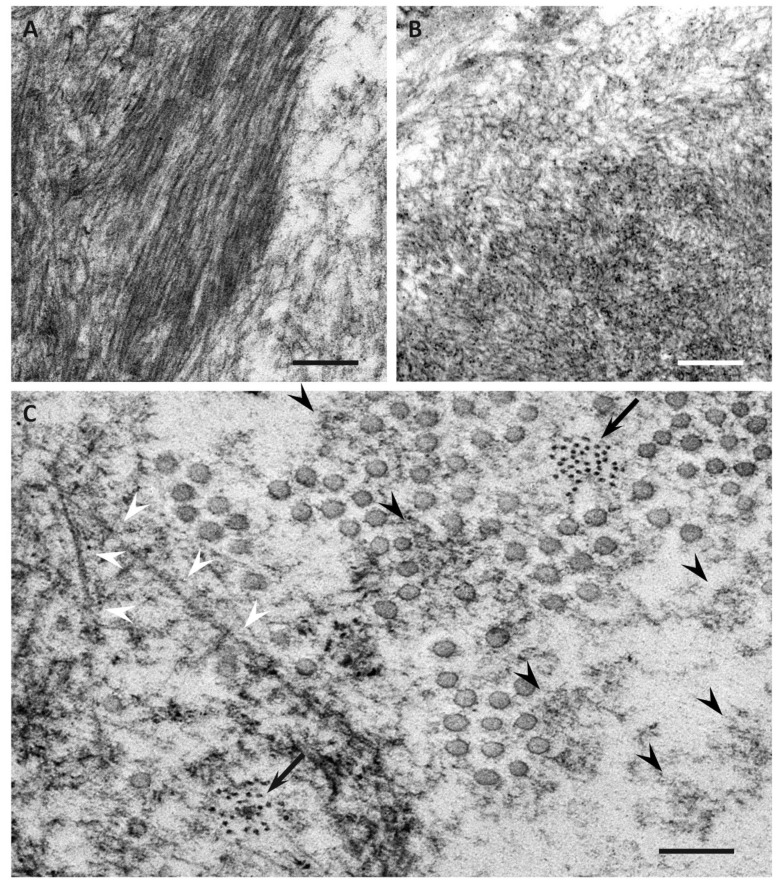
Representative electron microscopic photographs of amyloid fibrils in early-onset ATTR Val30Met amyloidosis patients from endemic foci (**A**,**C**) and late-onset ATTR Val30Met amyloidosis patients from nonendemic areas (**B**). Cross sections of sural nerve biopsy specimens. Uranyl acetate and lead citrate staining. Amyloid fibrils tend to be long and thick in early-onset patients from endemic foci (**A**), whereas those in late-onset patients from nonendemic areas are generally short and thin (**B**). Dotty structures (arrows) are frequently observed among amorphous electron-dense extracellular materials (black arrowheads) (**C**). Elongated, mature amyloid fibrils are also observed (white arrowheads). Circular structures with a diameter of 50 to 70 nm are collagen fibers. Scale bars = 0.2 μm.

Previous studies have demonstrated differences in the characteristics of amyloid fibrils depending on the age of onset and the type of mutation in patients with ATTRv amyloidosis [51,53,54,55,56]. In early-onset Val30Met cases, long and thick amyloid fibrils are common (Figure 2A), whereas the fibrils are usually short and thin in late-onset Val30Met cases and most non-Val30Met cases (Figure 2B) [51,54,56]. In addition, amyloid deposits in early-onset Val30Met cases tend to be highly congophilic and show strong apple-green birefringence, while those in late-onset Val30Met cases are generally weakly congophilic and show faint apple-green birefringence (Figure 1) [55]. These differences in the characteristics of amyloid deposits between early- and late-onset cases are particularly conspicuous in the heart [53,55]. Interestingly, short amyloid fibrils and a weak affinity of amyloid deposits for Congo red have also been reported for cardiac amyloid deposits in patients with ATTRwt amyloidosis [57]. A study of autopsied Japanese Val30Met patients demonstrated that most TTR in cardiac amyloid deposits from the early-onset cases was variant TTR, whereas wild-type TTR constituted more than half of the TTR in the deposits from the late-onset cases [55]. In ATTRv amyloidosis patients who undergo liver transplantation, cardiac amyloidosis may progress even after transplantation due to wild-type TTR deposition, particularly in elderly male patients [58,59]. These findings suggest that the mechanism of amyloid deposition in the heart is similar between late-onset ATTRv amyloidosis patients and ATTRwt amyloidosis patients. Interestingly, ATTRwt amyloidosis mainly affects males, who account for approximately 90% of patients [38,39]. This male preponderance is in accordance with late-onset ATTR Val30Met amyloidosis cases [10], but not with early-onset Val30Met cases, which show a nearly 1-to-1 male-to-female ratio [42].

An important issue tightly related to the contribution of wild-type TTR to the mechanisms of amyloid fibril formation is the truncation of TTR by proteases, such as trypsin and plasmin [32,33]. A large amount of C-terminal fragments of TTR, starting at positions around amino acid 50, have been found in the amyloid deposits of late-onset ATTR Val30Met amyloidosis cases and most ATTRv amyloidosis cases with non-Val30Met mutations, whereas N-terminal fragments are present in only small amounts [53,54,60]. C-terminal fragments are also present in the amyloid deposits of ATTRwt amyloidosis cases [57,60]. By contrast, amyloid deposits consist mainly of full-length TTR in early-onset Val30Met patients [53,60]. Importantly, truncated TTR resulting from proteolytic cleavage was shown in vitro to remain associated with the tetramer and was released only under certain circumstances, such as shear stress [61]. As organs liable to receive shear stress, such as the heart, ligaments, and tendons, tend to have amyloid deposits resulting from wild-type TTR deposition in elderly patients [62], TTR truncation may determine the sites of amyloid deposition, particularly in elderly patients.

## 6. Impact of Amyloid Fibril Formation on Neighboring Tissues

**Figure 3:**
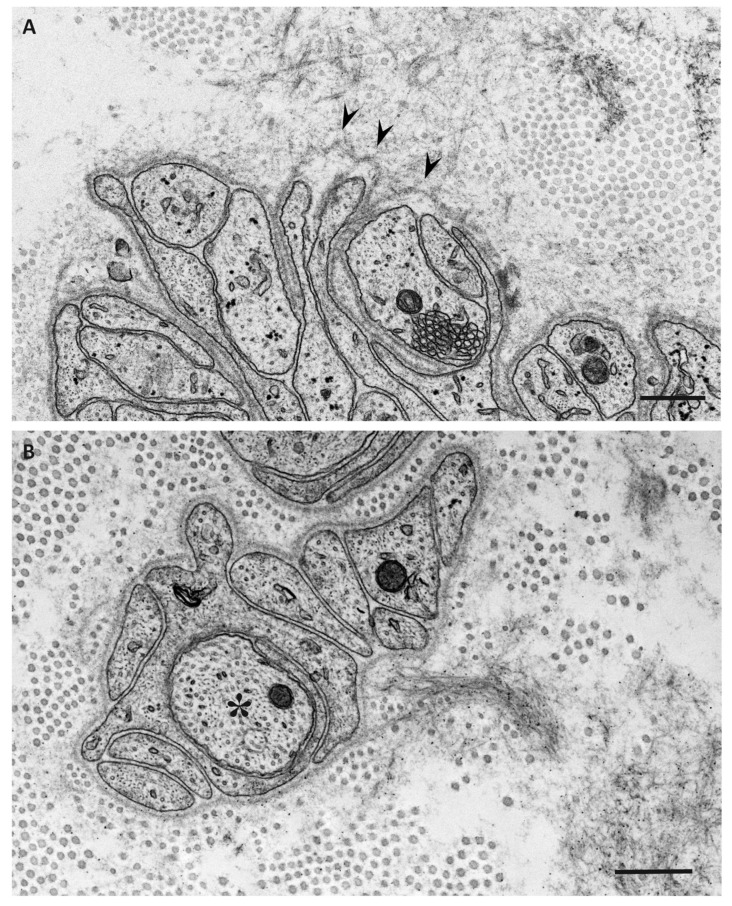
Impact of amyloid fibril formation on neighboring tissues in early-onset ATTR Val30Met amyloidosis patients from endemic foci (**A**) and late-onset ATTR Val30Met amyloidosis patients from nonendemic areas (**B**). Cross sections of sural nerve biopsy specimens. Uranyl acetate and lead citrate staining. During the process of amyloid fibril maturation, amyloid fibrils seem to pull surrounding tissues. This traction of neighboring tissues seems to be conspicuous in patients with long and thick amyloid fibrils, such as early-onset Val30Met patients from endemic foci (**A**). By contrast, the impact of amyloid fibril maturation on neighboring tissues seems to be less in patients with short and fine amyloid fibrils, such as late-onset Val30Met patients from nonendemic areas (**B**). The stretched basement membrane in (**A**) is indicated by arrowheads. An unmyelinated fiber in (**B**) is indicated by an asterisk. Scale bars = 0.5 μm.

Electron microscopic studies of nerve biopsy specimens from patients with ATTRv amyloidosis have shown that amyloid fibrils were formed among amorphous electron-dense materials located in extracellular spaces of the endoneurium [56]. Amorphous electron-dense materials tend to be observed around microvessels and the subperineurial space. Among these amorphous materials, dotty or fine fibrillar structures are frequently observed (Figure 2C). The dotty structures seem to be the core of amyloid fibrils because slightly elongated fibrillar structures with a thickness similar to the diameter of these dots are frequently found [56]. The mature long fibers usually occupy the central part of the large aggregations of amyloid fibrils, while the amorphous materials, dotty structures, and short amyloid fibrils tend to be present at the periphery of the aggregates of amyloid fibrils. During the process of amyloid fibril maturation, amyloid fibrils seem to pull surrounding tissues [56]. This traction of neighboring tissues seems to be conspicuous in cases with long and thick amyloid fibrils, such as early-onset Val30Met cases in endemic foci (Figure 3A) [51,56]. By contrast, amyloid fibril maturation seems to have a smaller influence on neighboring tissues in cases with short and fine amyloid fibrils, such as late-onset Val30Met cases in nonendemic areas (Figure 3B) [51,56].

**Figure 4:**
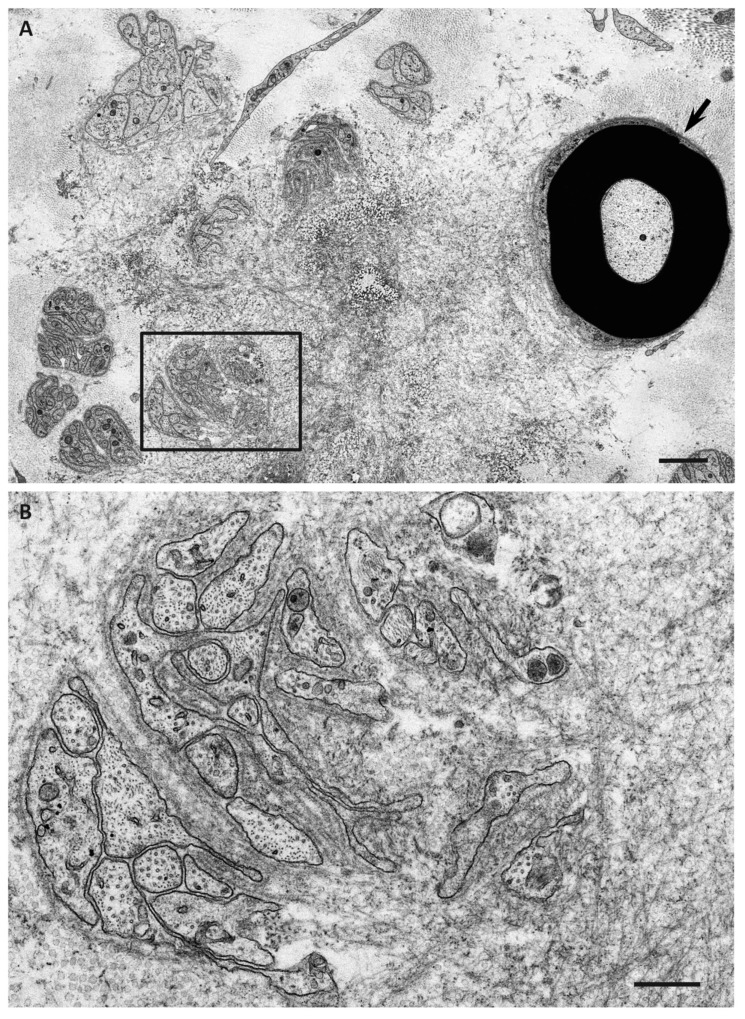
Aggregation of amyloid fibrils and Schwann cells in ATTRv amyloidosis. A cross section of sural nerve biopsy specimen from an early-onset Val30Met patient from an endemic focus. Uranyl acetate and lead citrate staining. Schwann cells associated with unmyelinated fibers that are apposed to amyloid fibrils become atrophic and distorted, whereas myelinated fibers, particularly large myelinated fibers (arrow), tend to be preserved because the apposition of these fibers to amyloid fibril aggregates is usually partial. A high-powered view of representative Schwann cells associated with unmyelinated fibers in the box in (**A**) is shown in (**B**). Scale bars = 2 μm (**A**) and 0.5 μm (**B**).

As a result, Schwann cells adjacent to amyloid fibril masses become atrophic and distorted, particularly in early-onset patients with long and thick amyloid fibrils (Figure 4) [51,56]. Small-diameter nerve fibers, particularly unmyelinated fibers, seem to be liable to this direct insult resulting from amyloid fibril formation. In contrast, myelinated fibers, particularly large myelinated fibers, seem to be resistant to such stress because the contact between these fibers and amyloid fibril aggregates is usually partial, even though the contact does occur. In addition, the basement and cytoplasmic membranes of Schwann cells that are apposed to amyloid fibrils, particularly long fibrils, tend to become indistinct, suggesting the direct damage of Schwann cells by amyloid fibril invasion [51,56]. An affinity of amyloid fibrils for Schwann cell membranes mediated by their common constituents may participate in this process [63]. A previous study suggested that TTR binds to the plasma membrane and exerts toxic effects by altering membrane fluidity [64]. 

## 7. Toxicity of Nonfibrillar TTR

There is accumulating evidence that the oligomers of amyloidogenic proteins play a key role in mediating toxicity in other common neurodegenerative diseases, including Alzheimer’s disease and Parkinson’s disease [65]. To support this view, a recent study using *Caenorhabditis elegans* expressing human TTR demonstrated the neurotoxicity of TTR oligomers [66]. In vitro studies using Schwannoma cell lines have also suggested the toxic effects of TTR on Schwann cells [67,68,69]. Interestingly, oligomers, rather than mature amyloid fibrils, seem to exert this toxic effect [67]. Hence, biochemical stresses may be responsible for Schwann cell damage in patients with ATTRv amyloidosis, in addition to the mechanical stress resulting from the formation of amyloid fibrils described earlier. Mechanical stress resulting from the direct effect of amyloid fibril elongation may explain the occurrence of small-fiber-predominant axonal loss that characterizes early-onset ATTR Val30Met amyloidosis patients [56]. In late-onset ATTR Val30Met amyloidosis patients, smaller amounts of amyloid deposits are found in the peripheral nervous system, even though the extent of nerve fiber loss is more severe than in early-onset ATTR Val30Met amyloidosis patients [34,51]. Hence, biochemical stress evoked by TTR oligomers may participate in the mechanisms of nerve fiber damage, particularly in late-onset ATTR Val30Met amyloidosis cases [34]. Indeed, TTR immunostaining-positive but Congo red-negative nonfibrillar precursors of amyloid (i.e., TTR oligomers) have been found in the endoneurium of ATTRv amyloidosis patients [34,67], even in the early stage of neuropathy [67]. Animal studies also demonstrated similar TTR oligomers in the peripheral nervous system [70,71]. An autopsy study suggested that TTR oligomers are more conspicuous in nerves from late- than early-onset patients [34]. 

## 8. Angiopathy Enhancing the Leakage of Circulating TTR

**Figure 5:**
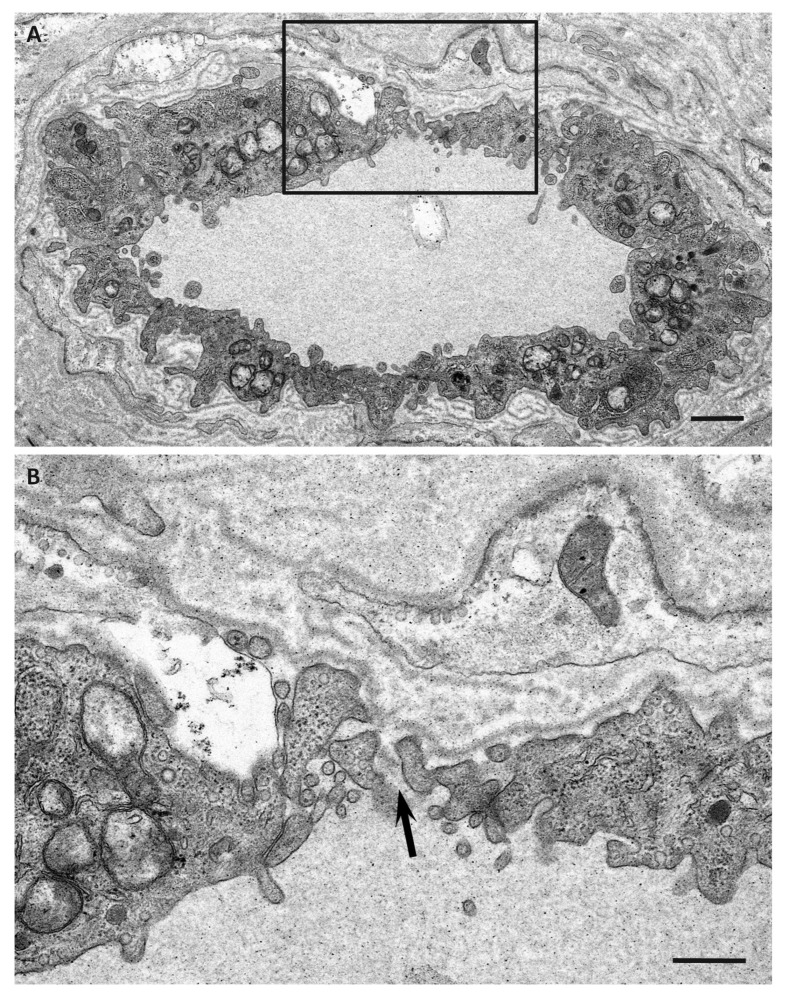
Microangiopathy in ATTRv amyloidosis. A cross section of sural nerve biopsy specimen from a late-onset Val30Met patient from a nonendemic area. Uranyl acetate and lead citrate staining. The continuity of endothelial cells of an endoneurial microvessel is lost (arrow), indicating the disruption of the blood–nerve barrier at this site. A high-powered view of the box in (**A**) is shown in (**B**). Scale bars = 1 μm (**A**) and 0.5 μm (**B**).

In addition to evidence regarding the toxicity of nonfibrillar TTR to the peripheral nervous system described earlier, recent studies suggested angiopathy as an early lesion enhancing the leakage of circulating TTR into extracellular spaces [51]. Cardiac magnetic resonance imaging revealed gadolinium enhancement in patients with ATTRv amyloidosis [72], suggesting that the leakage of serum components was enhanced in the setting of amyloid deposition. Retinal angiopathy has also been demonstrated in ATTRv amyloidosis patients [73]. Regarding the peripheral nervous system, extensive amyloid deposits completely surrounding the endoneurial microvessels have been shown as evidence of microangiopathy [74,75]. However, a recent study demonstrated the disruption of blood–nerve barriers of endoneurial microvessels, even in microvessels with scarce or no amyloid deposits around them (Figure 5) [51]. Studies using magnetic resonance neurography demonstrated a significant increase in the diameter of the nerve trunk in ATTRv amyloidosis patients, even in asymptomatic carriers [76]. This swelling of the nerve trunk may be at least partly attributable to the endoneurial edema associated with blood–nerve barrier disruption. A previous study suggested that variant TTR induces abnormalities in endothelial cells [77]. Recent studies performed from the standpoint of microangiopathy associated with diabetes mellitus also suggested that TTR primarily affects endothelial cells through apoptotic effects [78]. In addition, retinol-binding protein, which binds to TTR, influences endothelial cells via inflammatory activities [79]. Hence, it may be hypothesized that the endothelial cells, which are the front-line barrier of the peripheral nervous system to circulating variant TTR, are affected initially before the initiation of amyloid fibril formation. Notably, the disruption of blood–nerve barriers is more frequently observed in late-onset ATTRv amyloidosis patients with smaller amounts of amyloid deposits than in early-onset patients [51].

## 9. Therapeutic Insights from Pathology

As it is widely accepted that amyloid deposition causes tissue damage in ATTR amyloidosis, possible major therapeutic strategies for this disease consist of either a mixture of (1) reducing TTR production [16,20,21], (2) stabilizing TTR to prevent misfolding [18,19,20], or (3) eliminating already-deposited TTR [80]. Liver transplantation has been established as a treatment for ATTRv amyloidosis patients, particularly for early-onset patients, from the viewpoint of halting the production of variant TTR by the liver [16,17]. However, late-onset patients are not eligible for liver transplantation because the progression of cardiomyopathy and neuropathy continues, probably due to wild-type TTR deposition [58,81,82]. Hence, the introduction of noninvasive treatment by oral administration of TTR stabilizers, such as tafamidis and diflunisal, has had a great impact on the management of ATTRv amyloidosis [18,19]. The efficacy of TTR stabilizers has been suggested even for ATTRwt amyloidosis patients [20]. Epigallocatechin-3-gallate, a nonspecific inhibitor of amyloid fibril formation, also seems to be effective [83]. In addition, intravenous administration of a short interfering RNA (siRNA), patisiran, and subcutaneous administration of an antisense oligonucleotide (ASO), inotersen, have been shown to be effective in ATTRv amyloidosis patients [21,22]. As siRNA and ASO are able to significantly reduce the production of both wild-type and variant TTR in the liver, these treatments seem to be a suitable therapeutic option for ATTR amyloidosis. As described earlier, TTR seems to exert harmful effects even when fibrillar structures recognized as amyloid fibrils are not formed. As circulating variant TTR may induce microangiopathy, which plays a role as an initial lesion of organ damage [51], eliminating causative proteins is more reasonable than merely stabilizing the protein if the long-term tolerability of this strategy is confirmed.

An increase in therapeutic options necessitates early diagnosis and initiation of appropriate treatment, even in late-onset patients, before irreversible organ damage occurs. However, many patients with ATTR amyloidosis are still overlooked or misdiagnosed with other diseases [48,84]. For example, ATTRv amyloidosis patients with predominantly neuropathic symptoms but no apparent family history tend to be initially regarded as having chronic inflammatory demyelinating polyneuropathy (CIDP), because electrophysiological findings suggestive of demyelination may be concomitantly observed in addition to those suggestive of axonal degeneration [42,48]. Pathological findings suggestive of myelin destruction have been reported in patients with ATTRv amyloidosis, although these findings are rare [56,85]. Findings suggestive of subclinical cardiac amyloidosis, such as elevated plasma brain natriuretic peptide values, an increased cardiothoracic ratio on chest X-ray, a low voltage on electrocardiogram, and increased interventricular septal thickness on echocardiography, are important clues that suggest ATTRv amyloidosis in patients with neuropathy that mimics CIDP [10,48]. A study suggested that ATTR amyloidosis patients with predominantly cardiac symptoms constitute a significant proportion of patients exhibiting heart failure with preserved ejection fraction [52]. Therefore, progress in the treatment of ATTR amyloidosis will attract physicians’ attention to this disease, which manifests with a large variety of clinical presentations, leading to an increase in the number of newly diagnosed patients. Appropriate diagnosis and treatment programs for ATTR amyloidosis that account for the variability of this disease must be established. From this viewpoint, the long-term efficacy and tolerability of novel therapies, particularly siRNA and ASO, should be determined in the future.

## Figures and Tables

**Table 1:** Comparison of the two major forms of hereditary transthyretin Val30Met amyloidosis *.

Features	Early-Onset Patients from Endemic Foci	Late-Onset Patients from Nonendemic Areas
Age of onset	Late 20s to early 40s	≥50 years
Sex	Male = female	Male > female
Family history	Common	Frequently absent
Penetrance rate	High	Low
Cardiac involvement	Conduction defects	Heart failure
Sensory dissociation	Common	Rare
Autonomic dysfunction	Severe	Mild
in early disease stage		
Modality of nerve fiber loss	Small > large	Small = large
Amount of amyloid deposits	Large	Small
in the peripheral nervous system		
Length of amyloid fibrils	Long	Short

* Based on previous reports [2,34,51].
